# Memory-Related Encoding-Specificity Paradigm: Experimental Application to the Exercise Domain

**DOI:** 10.5964/ejop.v15i3.1767

**Published:** 2019-09-27

**Authors:** Danielle Yanes, Emily Frith, Paul D. Loprinzi

**Affiliations:** a Exercise & Memory Laboratory, Department of Health, Exercise Science and Recreation Management, The University of Mississippi, University, MS, USA; University of South Wales, United Kingdom

**Keywords:** acquisition, cognition, consolidation, encoding, episodic, physical activity

## Abstract

The Encoding-Specificity Paradigm indicates that memory recall will be superior when contextual factors are congruent between memory encoding and memory retrieval. However, unlike other contextual conditions (e.g., verbal context, mental operations, global feature context, mood dependency, and physical operations), this paradigm has nearly been ignored in the exercise domain. Thus, the purpose of this study was to examine the Encoding-Specificity Paradigm in the context of exercise and rest conditions. 24 young adults (age: M = 21 years) completed a within-subject, counterbalanced experiment involving four laboratory visits, including 1) R-R (rest-rest) condition, 2) R-E (rest-exercise) condition, 3) E-R (exercise-rest) condition, or 4) E-E (exercise-exercise) condition. The exercise bout included a 15-minute moderate-intensity walk on a treadmill. Memory recall was assessed via a 15 word-list task. Memory recall was greater for R-R (8.71 ± 3.1) versus R-E (7.46 ± 2.8), and similarly, for E-E (8.63 ± 2.7) versus E-R (8.21 ± 2.7). The mean word recall for the congruent and incongruent conditions, respectively, was 8.67 (2.4) and 7.83 (2.4). There was a statistically significant condition effect (F = 5.02; P = .03; partial η² = .18). This experiment provides direct support for the Encoding-Specificity Paradigm in the exercise domain.

The Encoding-Specificity Paradigm (Tulving & Thomson, 1973) indicates that memory recall will be enhanced when contextual factors are congruent between memory encoding and memory retrieval (e.g., studying and taking an exam in the same room). Although not conclusive across all studies (Fernandez & Glenberg, 1985), there is support for this paradigm across a multitude of domains. These domains, which will be discussed in the narrative that follows, includes mental operations, verbal context, global environmental context, global feature context, mood dependency, and physical operations. This has been thoroughly addressed elsewhere (Roediger III et al., 2017) and is further highlighted in the narrative that follows.

The transfer-appropriate processing model is an extension of the Encoding-Specificity Paradigm as it relates to broader *mental operations*. That is, if the cognitive processing at encoding matches that during retrieval, then memory retrieval will be enhanced. Types of processing can range from shallow levels-of-processing to more deeper levels (Craik & Lockhart, 1972). For example, and broadly speaking, techniques may include orthographic characteristics (e.g., focusing on the case of the letters in the word, such as upper or lower cases), phonemic characteristics (e.g., focusing on words it rhymes with), and semantic characteristics (e.g., meaning of the words). Moreover, Challis, Velichkovsky, and Craik (1996) have evaluated five related techniques, including 1) intentional learning (i.e., have participants learn as they see fit with no provision of instructions), 2) counting and reporting the number of letters while studying words (letter counting), 3) counting the number of syllables in the word (syllable counting), 4) making judgements about whether the word is living or non-living (living/non-living judgement; e.g., wooden board is non-living, tree is living), and 5) judging how relevant the words are to themselves (self-reference judgement). The latter and more deep-processing techniques are generally more superior in enhancing memory recall.

Regarding the *verbal context*, among Russian-English bilingual students, when prompt words were in Russian, participants recalled more Russian autobiographical memories, but when prompt words were in English, they recalled more autobiographical memories from their time in America (Marian & Neisser, 2000). Similarly, in Mandarin-English bilingual students, when questions were asked in Mandarin, participants responded more with answers coming from general knowledge acquired in Mandarin, and when the questions were asked in English, they responded more with answers from general knowledge that was acquired in English, suggesting that, perhaps, cognitive-search strategies at recall are dependent upon the language utilized at initial encoding (Marian & Kaushanskaya, 2007). These studies demonstrate support for the Encoding-Specificity Paradigm to autobiographical memory and semantic memory.

For the *global environmental context*, if environmental characteristics are similar for encoding and retrieval, then this may help to enhance memory retrieval. Godden and Baddeley (1975) instructed scuba divers to learn a list of unrelated words either underwater or on dry land and then recalled them later either in a matching or mismatching context. Notably, the number of words recalled was higher in the matching contexts (e.g., learning under water and retrieval under water) than in the mismatching contexts (e.g., learning under water and retrieval on dry land). Smith, Glenberg, and Bjork (1978) also showed similar results, but instead, had participants encode and retrieve in the same or different rooms. Interestingly, Smith (1979) also showed that being physically present in the previous environment was not a necessity for enhanced retention. Imagining the previous environment in which the material was studied was enough to enhance recall.

The *global feature context* is related to the *global environmental context*, however the former focuses on environmental features (e.g., the background screen color on the computer) while holding the physical context (e.g., the room the computer is in) constant. Grant et al. (1998) manipulated background noise of the room while keeping the room constant, and showed that the matching conditions enhanced memory recall more than a mismatching condition. Similarly, Geiselman and Glenny (1977) familiarized participants with male and female voices before the study session and told the participants to rehearse each visually presented word either in the male voice, female voice, or in their own voice for 10-seconds during the study session. Participants subsequently completed an auditorily recognition test, with words presented in either the male or female voice. When the voice of the word during the test matched the rehearsed voice (male-male or female-female), participants recognized the words better when compared to the mismatched condition. Clearly, the feature and environmental context plays an important role. Interestingly, research demonstrates that the context may not have to be exactly the same. For example, it may not be necessary to view the exact same baseball game on the television during encoding and retrieval. It is possible *any* baseball game could suffice. Several experiments demonstrate that when the reinstated context is similar, but not identical to the original context, then enhanced memory is still observed, but to a lesser degree (Smith, Handy, Angello, & Manzano, 2014).

Specifically, Smith et al., utilized a videotape exposure protocol, which had been employed in previous related research (Smith et al., 2014; Smith & Manzano, 2010). As noted previously, when participants viewed videos that were exactly congruent at both encoding and retrieval, memory recall was superior. However, as even similar contexts were evidenced to stimulate recall to a greater extent than dissimilar video scenes, the authors suggest that conceptual similarity between encoding and recall stimuli may offer robust support for a tendency to process similar environments by employing global methods (Smith et al., 2014). For example, even if the original environmental context featured a baseball game, a depiction of a softball or kickball game in a similar outdoor stadium or field may be coded via identical cognitive strategies. Although, importantly, the efficacy of global feature contexts in facilitating memory within this paradigm is governed by the “fit” between mental representations at encoding and retrieval. If a volleyball game and basketball game were represented similarly, as they both occur on indoor courts, a free throw would not match any aspect of the volleyball game itself. Thus, general cue representation at encoding may activate general environmental contexts at retrieval (e.g., encoding-court and retrieval-court); however, general and specific cues are not likely to confer benefits proposed by the Encoding-Specificity Paradigm (e.g., encoding-court and retrieval-three-point shot; Smith et al., 2014).

Regarding *mood dependency* (or state dependency), memory retrieval appears to be enhanced when mood is congruent during encoding and retrieval. Eich, Weingartner, Stillman, and Gillin (1975) had participants smoke a cigarette containing marijuana or a placebo cigarette before learning a list of words, with matching and mismatching occurring at retrieval 4-hours later. A mood dependency effect was observed. This effect has also been observed in other work using alcohol (Petersen, 1977) and caffeine (Kelemen & Creeley, 2003) to manipulate the participant’s pharmacological state. Other work also demonstrates this dependency effect (for multi-list learning) when manipulating mood via hypnosis (Bower, Monteiro, & Gilligan, 1978). Relatedly, and rather than inducing a particular mood via hypnosis, Eich, Macaulay, and Lam (1997) evaluated participants with bipolar disorder, and during the naturally occurring mood changes, they superimposed the experimental manipulations and confirmed this mood dependency effect among those who frequently experience mood shifts.

Of particular interest to our experiment (i.e., examining the Encoding-Specificity Paradigm in the exercise/physical domain), research demonstrates that integrating movement (*physical operations*) can help facilitate memory retrieval. Engelkamp, Zimmer, Mohr, and Sellen (1994) made participants either read phrases verbally (e.g., “close the book”) or read them and subsequently performed them (i.e., the participant closes a book in front of the researcher while reading the phrase “close the book”). When participants performed the action (“enactment effect”) at both the study session and the testing session, they recognized the phrases better than if they only performed them at the study session and read them at the testing session. Similarly, Dijkstra, Kaschak, and Zwaan (2007) observed that recollection of memories was enhanced when participants adopted body postures that were congruent with the autobiographical memories they were trying to retrieve.

Related to physical operations, but with more extensive physical movement, Thompson, Williams, L'Esperance, and Cornelius (2001) had participants learn 20 unrelated words either on land or in the air in parachutes and recalled them 8-minutes later in one of the two environments. When learning occurred on land, context dependency was observed. However, when learning occurred in the air, recall was poor for both the same and different context conditions. Researchers suggested that parachuting-induced stress may have impaired attention during encoding. In their next experiment, Thompson et al. (2001) used a video of skydiving instead of asking participants to skydive. Participants studied words through audiotape while watching a skydiving video or not, and from this experiment, context dependency was achieved.

In alignment with recent recommendations to evaluate memory retrieval within exercise contexts, specifically examining whether congruence of exercise during learning and recall influences accuracy of memory performances (e.g., rest-rest or exercise-exercise), and whether the specific environment (e.g., outdoor vs. indoor) may play an additional role (Loprinzi, Frith, Edwards, Sng, & Ashpole, 2017), the present study aims to extend our understanding of the Encoding-Specificity Paradigm by specifically evaluating its utility in the exercise domain. Unlike the other domains addressed above (mental operations, verbal context, global environmental context, global feature context, mood dependency, and physical operations), the application of this paradigm under ambulatory exercise conditions is relatively unknown. This is worthy of investigation, as not only will it provide further domain-specific context knowledge of the Encoding-Specificity Paradigm, but emerging work has started to examine the effects of acute exercise on memory retrieval (Crush & Loprinzi, 2017; Frith et al., 2017; Loprinzi, Edwards, & Frith, 2017; Loprinzi & Kane, 2015; Sng, Frith, & Loprinzi, 2018). This research demonstrates that acute exercise shortly before memory encoding can help to enhance memory retrieval during a rested (seated) state. We extend this emerging body of work by examining whether congruent exercise and resting conditions during encoding and retrieval is more advantageous in memory recall than mismatched conditions.

Despite considerable extant work demonstrating the effects of verbal context, global environmental context, global feature context, mood dependency, and physical operations couched within the Encoding Specificity Paradigm, a comprehensive exploration of encoding processes specifically within the context of verbal memory, and congruent versus incongruent physical exercise has yet to be conducted. Further, a wealth of research suggests physical movement is capable of effecting favorable outcomes across a variety of mental assessments, including creativity, executive functioning, and learning and memory. Assessing the effects of exercise versus rest at both encoding and retrieval, as well as mismatching these physical operations at encoding and retrieval, will facilitate a deeper understanding of the efficacy of the Encoding Specificity Paradigm. As episodic memories are, among other structures, often encoded via involvement of the left prefrontal cortex and later retrieved via involvement from the right prefrontal cortex and cerebellum, activation of the prefrontal and cerebellar structures with exercise may be a strategy for impacting both encoding and retrieval of episodic information. Thus, it is critical to differentially investigate whether exercise itself is capable of substantively influencing the quality of episodic memory performance, or whether merely maintaining a congruent bodily state at the time of encoding and retrieval is sufficient to elicit performance effects in line with the Encoding Specificity Paradigm. Thus, the purpose of this study was to evaluate the Encoding Specificity Paradigm in the context of exercise. We hypothesize that memory performance would be enhanced during congruent (vs. incongruent) states.

## Method

### Study Design and Participants

This study was approved by the authors’ institutional review board and all participants provided written consent prior to participation. The present experiment is a within-subject, counterbalanced (via Latin-squares) study design. Participants completed four separate visits, each occurring approximately 48–72 hours apart. Further details on these visits is described below. In brief, 24 participants (college students) were recruited via a non-probability sampling approach (classroom announcements and word-of-mouth). This sample size is consistent with our other related experimental work (Crush & Loprinzi, 2017; Frith et al., 2017; Loprinzi & Kane, 2015; Sng et al., 2018). Participants were ineligible for this study if they self-reported being a current smoker, had a concussion in the past 30 days, were pregnant, currently taking medication to regulate mood, took marijuana or other illicit substances in the past 2 days, or had a diagnosis of ADD/ADHD or a learning disability. Further, for any of the visits, if they exercised 5 hours prior to the visit or consumed caffeine 3 hours prior, the visit was rescheduled.

### Measures

#### Surveys

On the first visit, participants self-reported their moderate-to-vigorous physical activity (MVPA) (MVPA min/week) using the two-item Physical Activity Vitals Sign (PAVS) survey (Greenwood, Joy, & Stanford, 2010). At the beginning of each visit, to assess mood status, participants completed the State Trait Anxiety Inventory (STAI) (Metzger, 1976). For this mood survey, participants rated 20 items (e.g., I feel calm; I feel nervous) on a Likert scale (1, not at all; to 4, very much so). Notably, for all four visits, internal consistency, as measured by Cronbach’s alpha, was >.8.

#### Retrospective Memory

For each visit, participants listened to a recording of 15 non-semantically related words (from the Toronto Word Poll) via sound-reducing headphones, with the words delivered every 1.5 seconds. The list was played twice, with a 10-second break in between. Each word list was unique for the different visits.

#### Exercise

Researchers instructed participants to walk on a treadmill (Woodway treadmill) for 15 minutes and select an appropriate pace by saying “Please select a pace similar to one you would choose if you were late to class. Thus, it will not be a leisurely walk. Nor will it be a run.” The self-selected pace was maintained during the exercise bout (i.e., the speed did not vary). The same speed was used for each of the subsequent visits.

#### Exercise and Memory Procedures

Depending on the visit (counterbalanced), participants either performed the 1) R-R (rest-rest) condition, 2) R-E (rest-exercise) condition, 3) E-R (exercise-rest) condition, or 4) E-E (exercise-exercise) condition.

- The R-R condition involved resting for 2-minutes; listening to the word list; resting (sitting) for 20-minutes (“white noise” playing through headphones; no access to cell phone); and then memory recall of the words.
- The R-E condition involved resting for 2-minutes; listening to the word list; resting for 17-minutes (“white noise”); walking on treadmill for 3-minutes; and then memory recall during the last 30-seconds of the walk.
- The E-R condition involved resting for 2-minutes; walking on treadmill for 3-minutes; listening to the word list during the last 30-seconds of the walk; resting (sitting; “white noise”) for 20-minutes; and then memory recall of the words.
- The E-E condition involved resting for 2-minutes; commencement of treadmill walking; after 3-minutes of walking, listened to the word list (while continuing to walk); then continued walking for the next 17-minutes; and during the last 30-seconds of this 20-minute walk, memory recall of the words occurred.

#### Statistical Analysis

Statistical analyses were computed in SPSS (v. 24). A repeated measures ANOVA was employed, evaluating differences in memory recall across the congruent (R-R, E-E) and incongruent (R-E, E-R) conditions. Partial eta-squared ($\eta_{p}^{2}$) effect size estimates were calculated. Statistical significance was established as the arbitrary alpha level of 0.05.

## Results

Table 1 displays the characteristics of the analyzed sample.

**Table 1:** Characteristics of the Sample (N = 24)

Variable	Point Estimate	*SD*
Age, mean years	20.9	1.8
Gender, % Female	50	
Race, % non-Hispanic white	70.8	
Waist circumference, mean cm	91.7	11.0
MVPA, mean min/week	214.7	194.6

*Note.* MVPA = moderate-to-vigorous physical activity.

The mean age was approximately 21 years, with the sample equally distributed across gender. Table 2 displays the mood state and physiological (i.e., heart rate) scores across the four experimental conditions. There were no differences in mood state, resting heart rate, heart during encoding for E-R and E-E conditions, or treadmill speed across the four visits. As expected, heart rate during memory retrieval was higher for the R-E and E-E conditions compared to R-R and E-R.

**Table 2:** Exercise Indices Across the Experimental Visits (N = 24)

Variable	R-R		R-E		E-R		E-E		Test-Statistic			
	*M*	*SD*	*M*	*SD*	*M*	*SD*	*M*	*SD*	*F*	DF	*p*	$\eta_{p}^{2}$
STAI, mean	28.1	6.6	28.0	7.1	30.2	7.6	28.9	7.7	1.34		.29	.16
HR at baseline, mean bpm	76.8	10.4	76.6	9.1	75.2	8.8	75.6	8.5	0.25		.85	.03
HR at encoding, mean bpm	-	-	-	-	114.3	15.3	112.8	13.9	0.75		.39	.03
HR at retrieval, mean bpm	79.5	9.9	107.1	12.2	78.3	9.8	115.1	11.2	65.4		.001	.92
Speed, mean mph	-	-	3.48	0.5	3.38	0.1	3.37	0.1	1.0		.38	.08

*Note*. HR = heart rate; STAI = state trait anxiety inventory; R-R = rest-rest; R-E = rest-exercise; E-R = exercise-rest; E-E = exercise-exercise.

**Figure 1:**
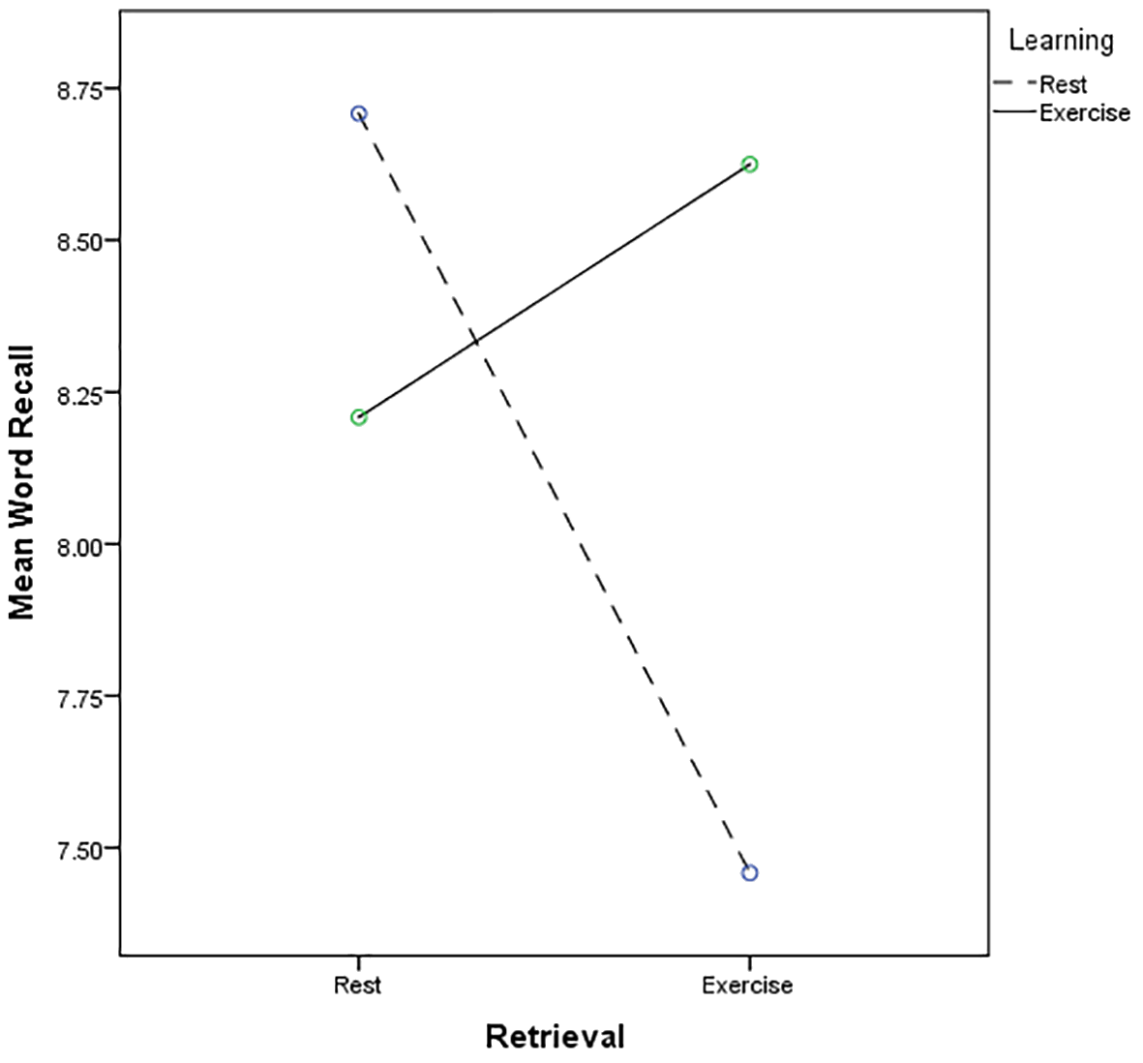
Mean number of words recalled across the 4-group encoding-specific paradigm.

In direct support of the Encoding-Specific Paradigm, memory recall was greater when there was congruence across the two conditions. That is, memory recall was greater for R-R (8.71 ± 3.1) versus R-E (7.46 ± 2.8), and similarly, for E-E (8.63 ± 2.7) versus E-R (8.21 ± 2.7). The mean word recall for the congruent and incongruent conditions, respectively, was 8.67 (2.4) and 7.83 (2.4). Indeed, there was a statistically significant condition effect (*F* = 5.02; *p* = .03; $\eta_{p}^{2}$ = .18). These results are displayed in Figure 1.

## Discussion

Unlike other domains (e.g., mental operations, verbal context, global environmental context, global feature context, mood dependency, and physical operations), the application of the Encoding-Specificity Paradigm in the exercise domain is much less investigated. The present experiment provides evidence that this paradigm also extends to ambulatory exercise. That is, matched conditions of rest-rest and exercise-exercise, were superior in enhancing memory recall when compared to mismatched conditions (rest-exercise and exercise-rest).

The present findings are in alignment with the few related studies on this topic. Miles and Hardman (1998) had participants (young adults) learn a list of 36 words during higher-intensity cycling exercise (heart rate > 130 bpm) or at rest. Identical to our findings (but our study employed a moderate-intensity exercise bout vs. high-intensity), they showed that recall did not differ for different encoding or retrieval conditions, but the interaction between them revealed a context-dependent effect. The only other related study on this topic was conducted by Schramke and Bauer (1997). In both younger and older adults, participants exercised (i.e., walked in a corridor) or rested for 5–7 minute before learning a word list. After a brief delay, participants engaged in the memory retrieval task, with context-dependency observed among both younger and older adults for free recall.

The present findings, coupled with those of Miles and Hardman (1998) and Schramke and Bauer (1997), suggest that the Encoding-Specificity Paradigm extends to the exercise domain. Importantly, to obtain a context-dependent effect, the context must be encoded during learning (i.e., part of the engram or memory trace) to be used during the retrieval phase. Thus, the retrieval cue must reactive a neural network pattern that is similar to the previously encoded pattern. Exercising or resting during encoding may create a unique engram pathway, and this unique neural network may be more likely to be reactivated if a similar context (e.g., exercise or rest) is present near/during the memory retrieval phase. As such, memories are not replicas of the actual event that took place, but rather, is a record of how we experienced the event. Our findings are not surprising as exercise versus rest conditions produce a much different experience during the development of the engram (memory encoding). In theory, the more contrasting the condition, the greater the likelihood that a context-dependent effect will be observed. This is supported by the seminal work of Godden and Baddeley (1975) who had participants learn a word list under water or on dry land (distinct conditions). Critically, though, the more events that are subsumed under the context, in theory, the context cue will be less effective in provoking the recall of the event/memory (cued overload principle) (Nairne, 2002; Tulving & Pearlstone, 1966). Applying this, it is uncertain if context-dependency will occur with all types and intensities of exercise. However, unlike the study by Miles and Hardman (1998) that employed a higher-intensity cycling protocol, we used a treadmill protocol of moderate intensity, with these differing contextual stimuli likely producing a differential magnitude of stimuli, yet both studies demonstrated a context-dependent effect. Although, at the same given intensity, and compared to cycling, treadmill exercise may induce a different neuroelectrical pattern given that treadmill exercise may require more attention and energetic constraint to maintain balance and control whole body coordination. It would be interesting to see if, at the same given intensity, whether open-skilled (i.e., activities that require the individual to react to unpredictable external stimuli) versus closed-skill exercises (walking/running) have a differential effect on the Encoding-Specificity Paradigm.

An interesting observation of our study was that the E-R condition had a higher memory performance than the R-E condition. Although additional work in this area is needed, this suggests that the timing of exercise may play a critical role in memory performance. In alignment with this observation, our other work has shown that when there is a close temporal coupling of exercise with memory encoding, memory performance is enhanced, when compared to other temporal periods (Haynes Iv, Frith, Sng, & Loprinzi, 2018). Another interesting observation of our study was that the R-R condition resulted in slightly higher (but not statistically significantly different) memory performance than the E-E condition. Such a pronounced effect is more likely to occur for a high-intensity bout of exercise, but this observation may, in part, be attributed to the transient hypofrontality effect (Dietrich, 2006; Dietrich & Sparling, 2004). That is, when encoding occurs during exercise, at higher intensities, encoding may be slightly compromised given that more metabolic and cognitive resources may be allocated to sustaining the movement.

Strengths of this study include the experimental design and examining the Encoding-Specificity Paradigm in the exercise domain, an under-investigated domain of this paradigm. It would have been interesting if we examined affective state (e.g., mood) not only prior to memory encoding (as we did), but also at memory retrieval, as this would have provided a clue as to whether our observed context-dependency was influenced by condition-related affective state. Previous research suggests that recall may be bolstered by congruence between encoding and retrieval relative to musically-induced mood (e.g., happy music-happy music; sad music-sad music) (Mead & Ball, 2007). As mood and arousal are suggested to play a potentially meaningful role in influencing the strength and durability of learning and memory processes (Bower et al., 1978), future work may wish to consider this possibility.

In conclusion, the present study provides direct support for the Encoding-Specificity Paradigm in the exercise domain. The main implications of this finding are the further support of this paradigm within a behavioral domain, as opposed to a health promotion implication of recommending exercise during memory retrieval (e.g., during a classroom exam), as the latter may be challenging and not practical from a logistical perspective. Nevertheless, non-exercise activity thermogenesis (NEAT) encompass locomotor and repetitive movement activities (Novak & Levine, 2007), such as fidgeting or squeezing a stress ball, that could plausibly be observed during test-taking, but the magnitude of effects may be largely dependent on individual-level factors. However, it would be worth evaluating whether this Encoding-Specificity Paradigm continues to apply to this behavioral domain with a slight modification to the temporal effects of bouted-exercise. That is, exercise immediately prior to (or even during) memory encoding, and rather than exercising during memory retrieval, have the exercise bout occur shortly before memory retrieval. If such a context-dependency effect is still observed, then this would have implications for promoting exercise immediately prior to taking an academic exam (while also exercising right before studying), for example. Research in this domain would also benefit by assessing other populations, such as older adults.
